# Unusual allergen in a butcher with respiratory symptoms 

**DOI:** 10.5414/ALX02126E

**Published:** 2020-12-02

**Authors:** Ingrid Sander, Claus Keller, Christina Czibor, Ursula Meurer, Rolf Merget, Monika Raulf

**Affiliations:** 1 Institute for Prevention and Occupational Medicine of the German Social Accident Insurance, Institute of the Ruhr University Bochum (IPA), Bochum, and; 2 Medical Practice for Pulmonary and Bronchial Medicine, Allergology and Environmental Medicine, Frankfurt am Main, Germany

**Keywords:** allergy, respiratory symptoms, butcher, transglutaminase, Streptomyces mobaraensis

## Abstract

A 37-year-old butcher developed respiratory symptoms during sausage and chicken production in a large company. In addition to various spices, the enzyme transglutaminase was a possible cause. The lung function test showed mild partial reversible airway obstruction and severe bronchial hyperresponsiveness. The IgE test showed sensitizations to various spice mixtures, coriander (0.74 kU/L), and to the ImmunoCAP-bound transglutaminase preparation from the workplace (7.12 kU/L). The skin prick tests with this transglutaminase were also positive. In the immunoblot of this preparation, a 40-kD protein reacted with the patient’s IgE and was identified as transglutaminase from *Streptomyces mobaraensis* by inhibition experiments. This is the first case of a butcher with an allergy to transglutaminase. After moving to a small enterprise without enzyme use, his symptoms improved. Sensitization and the course of the symptoms indicate a dominant role of transglutaminase in the patient’s allergic asthma.

**German version published in Allergologie, Vol. 43, No. 1/2020, pp. 15-19**

## Introduction 

While the risk of developing occupational allergic asthma is considered very high for bakers, this risk is considerably lower for butchers [2, 9]. In the scientific literature, there are only case reports of butchers with occupational asthma [3, 4, 5, 7, 14] or contact dermatitis [1, 16] caused by meat proteins, various vegetable spices, the red dye carmine, or the enzyme papain, which is used as a meat tenderizer. 

With this knowledge, a butcher who had developed respiratory problems while working in a large meat processing company was medically examined in Frankfurt in 2017. He had irritable cough and sneezing attacks in the morning during sausage production and the addition of spice mixtures with paprika and pepper. These symptoms became worse in the afternoon during chicken production, and he also suffered from shortness of breath and wheals on his skin. A possible trigger was the addition of very dusty binding agents which also contained the enzyme transglutaminase. The enzyme transglutaminase from *Streptomyces mobaraensis* catalyzes the cross-linking of proteins and thus leads to a more stable texture or joins pieces of meat. It has been increasingly used in the production of meat, fish, and dairy products since mid-2011. 

## Case report 

The 37-year-old butcher suffered from pollen allergy and bronchial asthma since his youth. He had been a smoker for 20 years with 10 cigarettes a day. For 8 years he has additionally suffered from season-independent symptoms during his work in the meat factory. These could not be prevented even when wearing a breathing mask, possibly due to insufficient tightness. However, he was only given the breathing mask after the symptoms began, and he did not wear it permanently. He relieved his severe respiratory symptoms in the afternoon during the production of chicken nuggets by double dosing his asthma spray containing beclomethasone dipropionate and formeterol fumarate (200 µg/6 µg per stroke). 

In the preliminary findings, there were indications of slightly elevated IgE antibodies to a spice mixture with laurel, paprika, green pepper, and mustard (test system unknown). The skin prick tests revealed sensitization to pollen from birch, alder, hazel, mugwort, and house dust mites. However, the total IgE value was within the normal range. In the documented lung function tests, a moderate obstruction (FEV_1_ 69% of the target value) was found, which was either not reversible, or could only be reduced by a maximum of 15% with β-mimetics. 

## Laboratory Analysis 

Two samples of the transglutaminase preparation from the workplace (manufacturer and composition unknown), and a transglutaminase from *Streptomyces mobaraensis* (Activa TI Ajinomoto, Modernist Pantry, USA) were analyzed. According to Modernist Pantry, Activa TI transglutaminase is an enzyme preparation without added helper protein for stabilization. 

**Figure 1.**
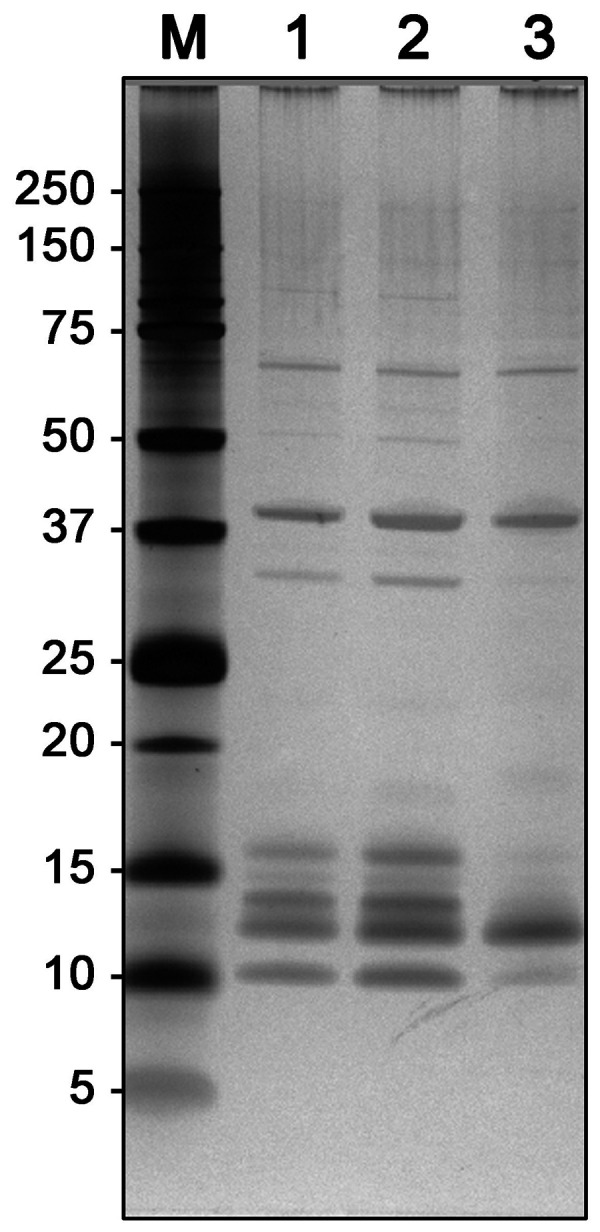
Silver-stained SDS-PAGE of transglutaminase preparations. M = molecular weight marker proteins, lane 1: transglutaminase from the workplace (May 2017), lane 2: transglutaminase from the workplace (June 2017), lane 3: Activa TI transglutaminase.

All samples were first analyzed by SDS-polyacrylamide gel electrophoresis (SDS-PAGE) and silver staining (Figure 1), with 0.5 µg protein per lane applied [10]. Both workplace samples had identical protein patterns with bands at 67, 40, 33, 16, 15, 13, 12, and 10 kDa (estimated average 22 kDa). The Activa transglutaminase showed silver-stained bands at 67, 40, and 12 kDa (estimated average 30 kDa). With a 5-fold molar excess of NHS-biotin, one transglutaminase from the workplace and the Activa transglutaminase were biotinylated and bound to streptavidin ImmunoCAPs [12]. Specific IgE binding was quantified using CAP-FEIA (Thermo Fisher Scientific, Phadia AB, Sweden). 

In addition, both transglutaminases (15 µg protein each) were transferred to PVDF membranes after SDS-PAGE, which were then cut into strips [10]. One strip each was stained with India Ink (1 h) [6]. The remaining strips were used for IgE immunoblots [10] with the patient serum and a control serum and IgE inhibition experiments. The control serum had a similar total IgE value and a comparable sx1 value (basic test inhalation allergens) compared to the patient’s serum. Each strip was incubated with 400 µL patient serum (or control serum), 80 µL 10% BSA (bovine serum albumin), and 320 µL phosphate salt buffer overnight at 4 °C; the inhibitor strips additionally contained 0.7% or 2.8% (w/v) of the transglutaminase preparations. 

## Results 

The patient’s lung function test in the body plethysmograph revealed a slight obstruction (FEV_1_ at 74% of target) and a highly hyperreactive bronchial system. 

The skin prick test with transglutaminase from the workplace was positive (wheal as large as in the positive control with histamine). In vitro sensitization was shown in the ImmunoCAP IgE test for coriander (f317, 0.74 kU/l) as well as for the workplace transglutaminase bound to ImmunoCAPs (7.12 kU/l) and Activa transglutaminase from *Streptomyces mobaraensis* (7.48 kU/l). The spices mace (f266), nutmeg (f282), cardamom (f267), and bell pepper (f218) were negative in the ImmunoCAP IgE test. The total IgE value was 55.9 kU/l. 

**Figure 2.**
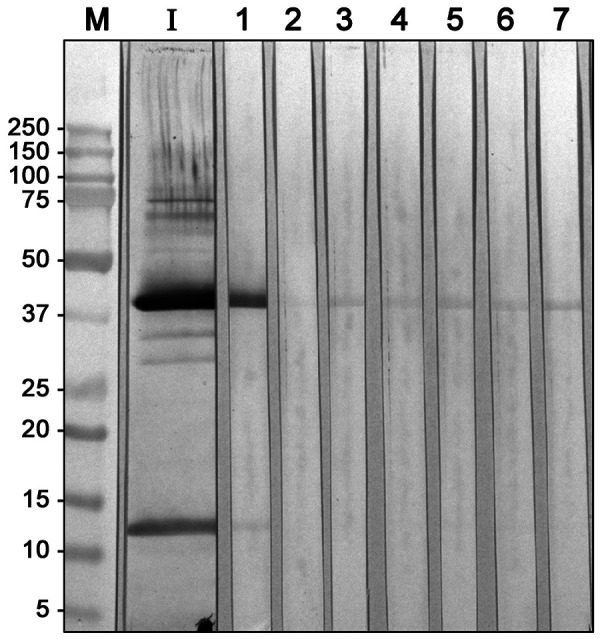
IgE immunoblot of transglutaminase from the workplace including inhibitions. M = molecular weight marker proteins, I = India-Ink staining of transglutaminase from the workplace (May 2017) on the blot membrane, lane 1: IgE binding of patient‘s serum, lane 2: reaction with control serum, lane 3: reaction of patient‘s serum inhibited with 2.8% Activa transglutaminase, lane 4: reaction of patient‘s serum inhibited with 0.7% Activa transglutaminase, lane 5: reaction of patient‘s serum inhibited with 2.8% transglutaminase from the workplace, track 6: Reaction patient serum inhibited with 0.7% transglutaminase from the workplace, lane 7: control with anti-IgE detection antibody.

In the immunoblot, a 40-kD protein was identified as IgE-binding, which was present in the preparation from the workplace as well as in the Activa enzyme preparation. Figure 2 shows the immunoblot with the workplace transglutaminase; the immunoblot with the Activa transglutaminase looks almost identical (not shown). The IgE binding of the patient serum to the 40-kDa protein could be completely inhibited with the workplace transglutaminase as well as with the Activa transglutaminase already at a concentration of 0.7%. The band at 40 kDa, which is still weakly recognizable after inhibition, has an intensity similar to that of the strips with the control serum or with the anti-IgE detection antibody. 

The patient reacted positively in both the nasal and bronchial provocation tests after workplace simulation with paprika powder. Provocation with transglutaminase was not performed for safety reasons, due to the reactivity of this enzyme. 

## Discussion 

With pre-existing pollen allergy, the butcher presented in this case report developed respiratory problems at work throughout the year. His symptoms were particularly severe in the afternoon while making chicken nuggets. In this process, the enzyme transglutaminase acts as a binder, linking pieces of meat by cross-linking proteins. Both the skin prick test and the specific IgE test showed a clear sensitization to the enzyme product used at the workplace. However, the composition of the enzyme product was unknown. In principle, sensitization to components added for stabilization, such as wheat or milk proteins, would also be possible. Therefore, an enzyme preparation with a known composition was additionally tested. This contained transglutaminase from the bacterium *Streptomyces mobaraensis* and no helper proteins. The sIgE test with this product was also clearly positive (slightly higher than with transglutaminase from the workplace). In the analysis by SDS-PAGE, the two transglutaminases differed only by some additional bands in the product used at the workplace. The IgE immunoblot then identified a 40-kDa protein in both products as the allergen. This fits well with the theoretical molecular weight of 44 kDa of the transglutaminase from *Streptomyces mobaraensis* [15]. The identity of the 40-kDa proteins of both products is supported by the complete inhibition of IgE binding of the patient’s serum to this protein band. 

The enzyme transglutaminase from *Streptomyces mobaraensis* is used in the production of meat, fish, and dairy products. Two previous cases have been described in the literature: a case of respiratory allergy to microbial transglutaminase in a person working in company where ingredients for the food industry were mixed together [11] and a case of the chef of a restaurant offering “molecular cuisine” who had developed urticaria, rhinoconjunctivitis, and asthma when using transglutaminase powder [8]. This is the first case of a butcher with an allergy to transglutaminase. After moving to a small enterprise where no enzymes are used, his symptoms improved. Sensitization and the course of the symptoms indicate a dominant role of transglutaminase in the patient’s allergic asthma. Transglutaminase from *Streptomyces mobaraensis* is therefore added to the list of industrially produced enzymes that are triggers of occupational respiratory allergies [13]. 

## Funding 

The study was financed by the German Social Accident Insurance (project IPA-14). 

## Conflict of interest 

The authors state that there is no conflict of interest.
